# A World Through Glass: A Narrative Around the Family Experiences During the Confinement of COVID-19

**DOI:** 10.1177/0192513X211024989

**Published:** 2022-06

**Authors:** Gustavo González-Calvo, Marta Arias-Carballal

**Affiliations:** 1 Departamento de Didáctica de la Expresión Musical, Plástica y Corporal, 16782 Universidad de Valladolid, Palencia, Spain; 2 Departamento de Inglés, Instituto de Enseñanza Secundaria “José Jiménez Lozano”, Valladolid, Spain

**Keywords:** parents, children, COVID-19, lockdown, social distancing

## Abstract

COVID-19 was declared a pandemic in March 2020, and the world has witnessed significant changes since then. Spain has been forced to go into extreme lockdown, cancelling all school classes and outdoor activities for children. Our study explores how parents of a group of school children aged 7 to 8 years have experienced confinement due to the COVID-19 health crisis. Following a narrative methodology, the results have been organized around a story that takes as a reference the period of confinement for a mother and worker in times of confinement. The conclusions of our study suggest that participants have experienced significant changes in their routines, having faced numerous personal and professional dilemmas in a climate of great emotional burden. This study is the first of its kind in investigating how the COVID-19 pandemic has influenced the ways that children and their families live and its possible implications for their futures.

“In every story there is always, in my opinion, some silence, some hidden gaze, a word that is silenced. Until we have given expression to the ineffable we have not reached the heart of history” (Coetzee, 2004, p. 136)

## Introduction

In December 2019, the first case of the so-called COVID-19 disease was registered in China (COronaVIrus + Disease + 2019). In May, the World Health Organization decided to declare it a global pandemic because of the rapid spread of the virus among the population and the many deaths it causes. That same month, Spain decreed a state of alarm, confining the entire national population to their homes for a period of 15 days (Royal Decree 463/2020 of March 14) and extended it on numerous occasions.

The first investigations into the consequences of the confinement soon began to appear. Thus, Brooks, Webster, and Smith (2020) and Brooks, Webster, Smith, and Woodland (2020) study the psychological impact caused by confinement, analyzing studies in different countries with people quarantined for different diseases, including Ebola, influenza, Middle Eastern respiratory syndrome and SARS-CoV-1 (a predecessor of the current COVID-19). The consequences of confinement include negative psychological effects, such as post-traumatic stress, confusion, irritability, fear of infection and anger at health and government policies. Other studies such as those by Jiang, Nan, Lv, and Yang (2020) show that youth, women and people with responsibilities towards others were more vulnerable to post-traumatic stress symptoms. In Italy, Chiara et al. (2020), in a study of 786 children aged 8 to 18, found that up to 30% of the participants were at high risk of post-traumatic stress. And in Spain, a transversal study that analyzes the symptoms of anxiety, depression and stress in a total of 3550 adults, reflects that confinement has a notable emotional impact, being the most predominant symptomatology among women, minors, people with symptoms compatible with COVID-19, people in psychological treatment and people with negative personal relationships within their family life (Odriozola-González et al., 2020). In a short time, the entire social, educational and economic system had to be reorganized to respond to the needs of the population. However, the needs of the child population seem not to have been adequately addressed. As an example, the law has allowed domestic dogs to be taken out for a walk, but not for minors to go out in the street. This situation, among others, leads us to think that during the confinement (that in the last days is becoming more flexible, allowing the minors to go out to the street under certain conditions), the socio-affective and psychological needs of the youngest have been obviated, with consequences that we still do not know.

Confinement has brought anxiety, stress and depression among the population (Wang, Zhang, Zhao, Zhang, & Jiang, 2020), and children are not exempt from this. According to Ghosh, Dubey, Chatterjee, and Dubey (2020), children are more vulnerable to the psychosocial impact of the pandemic. Given the closure of schools and the lack of physical activity outdoors, children are disrupted in their routines, and this can lead to monotony, distress, impatience, disturbance and various neuropsychiatric symptoms (Chiara et al., 2020; Odriozola-González et al., 2020). The most common feelings and emotions among children during this time of confinement have been anguish, worry, helplessness and fear (Saurabh & Ranjan, 2020). The youngest children (3–6 years) have expressed the most emotions, but in all age groups there has been evidence of clinging, inattention and irritability (Jiao et al., 2020). One of the possible causes of this tension may be the distress of parents who need to balance personal life, work and parenting (Spinelli, Lionetti, Pastore, & Fasolo, 2020).

The goal of our research is to explore how children have experienced and lived through this time of confinement as a consequence of the current COVID-19 pandemic as seen from their parents’ perspective. Specifically, we look at how family members of the children have perceived the time of confinement and how the health crisis has affected their daily lives. As far as we know, this work is the first of its kind to investigate how the COVID-19 pandemic has influenced the way of being and thinking of the minors, seen through the eyes of their closest relatives.

## COVID-19 and Childhood

The global COVID-19 pandemic is a stressor that originated outside the family system, but the novelty and uncertainty associated with this disease makes it likely that it will be perceived as a major stressor by many parents and children. According to Chung et al., 2020, the perceived parental impact of COVID-19 is associated with increased parenting stress and, in turn, increased risk of severe parenting. These factors are compounded by the economic uncertainty that many families go through and will go through, which contributes to emotional distress and increased risk of psychological problems in the short term (Pfefferbaum & North, 2020).

These unprecedented times have led to the imposition of social restrictions that negatively impact some groups far more than others. A spotlight has been shone on a wide range of social inequities and injustices, many of which directly affect children and young people. For example, although it appears that children’s physical health is less likely to be severely impacted by COVID-19, it is becoming more evident that current social conditions are having negative effects on children’s mental health, education and well-being. Questions such as whether they are as contagious as adults, whether they are the main transmitters of the disease, whether their immune system is more prepared than that of adults to overcome the disease and whether schools should be reopened as soon as possible, among others, represent an obvious uncertainty in which making decisions is not easy. Thus, from the beginning of the pandemic it was stated that children transmitted the disease more easily, that they were a vulnerable band and that a spiral of guilt entered the youngest. The children were the first to have to abandon their routines and, possibly, they will be the last to recover them once we return to a situation of certain normality, seeing their rights more diminished than other strips of citizenship.

Although measures taken by individual countries around confinement may be considered necessary, there is concern that prolonged home confinement and school closures, among others, could have negative effects on children’s physical and mental health (Brooks, Webster, and Smith (2020); Brooks, Webster, Smith, and Woodland (2020); Wang et al., 2020). These negative health effects are likely to be much worse when children are confined to their homes with no outdoor activities and no interaction with peers during the outbreak (Brooks, Webster, and Smith (2020); Brooks, Webster, Smith, and Woodland (2020)). In any case, the new situation we are experiencing is associated with a huge transformation in our lifestyle. It seems that nothing will be as it was before the emergence of COVID-19.

This new scenario, marked by fear of the body and physical proximity, will change the way children relate to others and, with it, their whole lives. As an example, teamwork, very common in the early educational and life stages of children, which has as its main objective to help in the socialization of the child, will be much more difficult in the day-to-day life of the pandemic since children will not be allowed to be together. The future of the relationships does not seem to be encouraging, exalting a model of ‘every man for himself’ represented by some when it comes to collecting basic necessities over a model of cooperation and solidarity. A world that is isolated, distrustful and selfish, or a world in which it is possible to understand the connection and need that all of us have, is opening up to the smallest of people.

On the other hand, among the measures that will be adopted at the beginning of the next school year, there are two that call for special attention: the replacement of the pavilions and gymnasiums where the school’s physical education is usually carried out and the school libraries by classrooms where the academic contents are followed, with the intention of fitting out more spaces that allow the safety separation of the students. It seems that educational policies do not take into account that Spain, according to the General Council of Medical Associations, is the fourth country in Europe with the highest rate of child obesity and the third European country that spends the least on reading. Once again, health concerns may take precedence over other rights considered essential for minors.

We cannot stop asking ourselves the question of ‘who will pay for the health crisis’. Other times throughout history, it seems to have been the poor, the weakest; it is possible that in this situation, among the weakest, those who have no voice, we must include children. Be that as it may, the new situation we humans are currently experiencing due to the current COVID-19 pandemic brings about significant changes in our lifestyles. These changes are evident with the return of some children to school, where in some countries there are now circles painted on the floor to define and limit the personal spaces of each student. Denial of affectionate gestures such as hugs and kisses is also common now, as well as other restrictions such as not being able to share toys and wearing surgical masks. The life of a child in Spain before the pandemic was significantly different than the current lifestyle. Children used to have plenty of extracurricular activities and socialising time with their friends. Even though outdoor parks have been emptier in the last few years, possibly as a consequence of children having so many extracurricular activities, they were not completely empty as they are now. Other children did not represent a threat for them. They were not ‘people to avoid’ for being afraid of contracting the virus, as it is today. Children were used to demonstrate their affection with close proximity and involving gestures such as kisses and hugs. Schools, families and extracurricular activities were places in which children could trust others and feel safe. To better understand this, in the study we present, we look in depth at the experiences, fears and expectations that a group of school children between the ages of seven and eight have had, seen from the perspectives of their parents.

## Methodology

This study uses narrative analysis to answer the research questions. Since the 1980s, scholars have begun to truly appreciate how people structure their experiences through the stories they tell, understanding that a person is essentially a storytelling animal (Frank, 2004; Smith & Sparkes, 2009). Smith and Sparkes (2009) explain that narrative, as a research method, is a complex genre that must contain certain elements: (a) a starting point, (b) protagonists and (c) the ability to connect events sequentially in time and space to give an explanation or consequence to the plot. The use of stories to make sense of experiences is well established within the realm of qualitative research (Riessman, 2008) and also helps to understand the relational and cultural fabric of human lives (Frank, 2010).

The criteria used to judge the quality and relevance of research answer the following questions: Is there anything worth learning from this story? Does it help to bring about a change in educational, social and family policies (e.g. reconciliation of family and work)? Finally, does it lead us to think about the consequences, values and moral dilemmas faced by families in a health crisis situation such as the one we are facing?

### Participants

The participants in this study were a group of 73 families of schoolchildren who were studying the second year of primary education in a state school in Spain. The socio-cultural and economic level of the participants' families can be considered average, their main occupations being automotive workers, service sector workers, construction employees and civil servants. It is important to note that several of the participants live in a single-family house, with more space and a small garden that favours the conditions of confinement.

The participants were asked to answer the following questions on a weekly basis from the time the confinement began in March until the end of the school year in June 2020: How is your child experiencing this period of confinement? What are the main difficulties you are facing? Do you feel that the confinement is having any positive aspects? Do you miss anything in your former family life?

The families of the minors were asked for explicit permission to reproduce in the research the answers to the questions requested. No names have been used in this article to preserve the confidentiality of all participants.

### Data collection

The families of the school children were asked to respond to the above-mentioned questions on a weekly basis and electronically for a total of 14 weeks. The authors transcribed their answers into a written story form. Story writing was chosen by the authors and participants with the belief that this form would be more appealing to the audience who would engage with it. Once the story representations were constructed in written form, the main ideas provided by the participants were synthesized into a single story. The story was sent to each participant for approval, addition or modification.

### Narrative analysis

The data were analyzed in narrative terms. Narrative analysis of the transcripts was carried out through interpretation of what was said (Riessman, 1993). Initially, this led to the identification of the main themes. The themes that emerged in the different interviews were grouped around different categories, as reflected in Table 1. From these categories, the story was developed.

**Table 1.** Categories arising from the questionnaires of the family members.

Discomfort/well-being
Confinement issues
Emotional management
Emotional footprint
Learning and ‘new normality’
Evolution, progressive adaptation and ‘ups and downs’
Work, telework and homework
Family issues
Resources to encourage and entertain
Spaces and their possibilities
Generation of social and relational moments
School as a place to socialize and meet friends
Rigour with the regulations: The ‘little police officers of COVID-19’

Subsequently, the authors undertook a second round of analysis through a process of constant comparison of the texts in order to establish credible and reliable sub-themes in each category (Gubrium & Holstein, 2009). Finally, relevant citations were selected to illustrate these themes and sub-themes with which the story was finally developed.

## Results

The following narrative recreates a story that takes as its protagonist a mother who, teleworking during the time of the pandemic, faces a series of dilemmas, problems and experiences as a result of COVID-19. We decided to recreate the story from a mother’s point of view because there were 71 mothers who responded to the study (out of a total of 73 participants). In this sense, they admitted they were very stressed as they had to work (usually telework) and look after the children at the same time, which included developing and executing lesson plans for their children and finding ways to support relatives and friends who may be in even more challenging straits than themselves.

We believe that this is a fiction that tells of a situation that can be assumed by most of the family members who have experienced a change in their lifestyle as a result of the health crisis. In this respect, in the conversations we have had with the participants from the elaborated story, they claim to feel well represented. The text, in italics, tells the story we have elaborated around the life of this mother during the confinement in an attempt to represent the voices of all the participants in the study.

*“Good afternoon, dear countrymen. Today I have just informed the Head of State that tomorrow an extraordinary council of ministers will be held to decree a state of alarm throughout our country, throughout Spain, for the next 15 days [...] We are only in the first phase of a fight against the virus being waged by all the countries of the world [...] As I said at the beginning of the week, very hard weeks await us [...] It cannot be ruled out that in the coming week, we will unfortunately reach more than 10. 000 affected [...] I also want to convey a very special message to our elderly and to people with chronic diseases that logically weaken their defenses: they must protect themselves as much as possible against infection, and avoid at all costs contact and exposure in public spaces. I would also like to address the young people who also have a decisive mission. It is true that because of their vitality they can feel sheltered from the most severe effects of the virus, but they can act as transmitters to others close to them who are much more vulnerable. Their collaboration, the collaboration of young people, is decisive in cutting off contagion and so they must limit contacts and maintain social distance [...]. [...] It’s going to be very hard and difficult, but we're going to stop the virus, that’s for sure. With unity, with responsibility and with social discipline”.*

Pedro Sánchez’s deep voice echoed through the screen as we held our breath, without looking at each other. Unaware of the announcement, the children continued to play quietly in another corner of the room. When the speech was over, I remember thinking: what now? I felt the bitter taste of attending a unique historical event in my mouth, but this privilege was difficult to digest along with the fear it aroused. I got up slowly, without saying a word, and looked out of the window. I guess I was instinctively hoping to find some obvious physical change in the world around me, something tangible that would prove that what we had just heard was not a bad dream. And there it was. Just a few minutes after the state of alarm had been made official and the Prime Minister had issued a long list of bans, things were beginning to change. The park in front of us was cordoned off with red and white tape. From that moment on it could not be used until... until when? I turned to look at my children. They were still playing in the same corner even though they had started to fight. What now? As if in the background, my husband was still watching the television. Again, I looked out the window. There was no one in the street. That was our world now. A world through glass.

It was already dinner time. As if we had agreed previously, my husband and I began an overreaction of jokes, stories and antics in that self-protective ‘nothing happens here’ tone to make our two children, who were certainly not yet aware of what had just happened, believe that this was just any Saturday night.

Later, under the silent complicity of a sleeping house, my husband and I finally talked about what had happened: perhaps this brutal change that is coming will bring more positive things than negative ones; perhaps things will not change much. ‘I do have to go out to work’, my husband said, ‘and, in your case, if teleworking is a challenge, you can take it as something to learn from. In principle it will only be 15 days. The important thing is that the children are well.’ The important thing was that the children were well.

With that conviction, I woke up the next morning, taken by an excess of energy that was intended to calm my particularly checkered mind, making it believe that everything was under control. I had carefully organized the time (work time, children’s ‘study’ time and leisure time). The telephone was burning with messages proposing what to do these days: shows, libraries, music, cinema, theatre... openly, to help people get through a quarantine that had just begun. It feels like we are going on holiday, I remember thinking, a bit upset. By the time a week has passed, we will have exhausted all suggestions and will not know what to do to encourage each other.

I tried to get away from these ideas. Once the children had had breakfast, I put the little one in the school babi (in line with the ‘nothing happens here and this is the most normal’ philosophy) and sat them both down at the dining room table to try and carry out the ‘school’ routine while their father went out to work. As soon as I opened the mail, I realized how illusive my plans were. I had several messages from each of the teachers with an endless list of suggestions and proposals. I felt overwhelmed. The little one is only in first grade, I thought. What is all this? It took me a long time to read it and find out what the children had to do while they looked at me expectantly and with a certain excitement. Given the ages of my children, the completion of tasks depended on my almost continuous presence and support. And how can I cope with this, the housework and my own work?

As the days went by, I was surprised by the maturity of my children to assimilate, naturalize and understand what was happening. The situation had already been explained to them at school, and we made up a little story so that they would understand what was happening. They soon accepted that they could not go out because of the coronavirus, except for Dad who was an ‘essential’ worker, something like a superhero in their heads. The first day they saw him come into the house half-naked when he returned from work (he undressed in the garage and immediately put his clothes in the washing machine, as recommended) and run to the shower after a very brief greeting, they looked at me in confusion looking for an answer, and on hearing my explanation, they started to laugh.

In general, there were no complaints, no fears and no excessive questions. Sometimes the little one would let his little concerns show. ‘Can we celebrate Halloween, mum?’, he asked. ‘What if the Three Kings can’t come because of the coronavirus?’ His fears would reflect in those terrible night terrors that had become more frequent. The eldest also started sneaking into our bed again, which automatically sent my husband to another spare room. Only there did he manage to have a peaceful sleep. Except for that kind of night catharsis that revealed that not everything ‘went great’, the days passed for them between joy and fun. Fortunately, we had a garden that allowed us to get some fresh air and have a slight, very slight, feeling of freedom. By virtue of being together and sharing spaces, the relationship between my two children was strengthened. Both of them missed their friends, and video conferences were a poor substitute for that human contact they so badly needed, so they looked to each other for the support and complicity they had previously found in their group of friends. Fights were frequent, but they also seemed to have developed their own mechanisms for resolving small conflicts. My husband and I worked hard to provide activities that would keep them entertained and we started to set up little routines: physical activity circuits in the garden, after-dinner board games and Saturday afternoon popcorn movies. Mornings were a bit different since we did not have to rush so much after waking up. There was no need to hurry to have breakfast, get dressed, brush our teeth and all those usual routines, so the kids got used to come to my bedroom and enjoy some playing time before actually getting up. We would imagine that the bed was a big raft and that we were fighting against a relentless sea, or we laughed our heads off while tickling each other. One of these mornings, my eldest son suddenly stopped the game, looked gravely at me, and admitted that he was happier now because we had a lot of family time. That innocent comment made me feel a twinge of guilt and anger: reconciling this family life with my new work life (with the total availability that teleworking implied) was providing me with extra, unhealthy stress. I was reassured to hear that the nervousness generated in me by this stress, which sometimes translated into an uncontrollable loss of patience with the children, was not perceived by them as an incomprehensible transformation of their mother into a monster.

I could not imagine how other families were doing it. Just the day before, another mother in the group had told us that her husband, a nurse by profession, had become infected. He would almost certainly have to spend several weeks in hospital and they could not see him or even talk to him. Having to wait for a daily call from an assistant at three o’clock in the afternoon to find out about their father’s condition was especially hard on the two daughters they had. Other parents had chosen to take their children to the village with their grandparents, where they were delighted in a free and healthy environment although far from their parents. What really fascinated me, however, was not hearing a single complaint from that co-worker with four children who lived in a not very large flat and claimed to get on well. We were not really close and yet I could not stop thinking about her, wondering and worrying a bit about whether she was being able to cope with everything. I think I even felt a bit sorry for the kids. That is why it really surprised me when she said: ‘The children keep each other company. They always find something to amuse themselves with. I think that the day they will be finally allowed to go out they will not want to’. ‘But, what about sunlight, friends, fresh air, the feeling of freedom?’—said I. ‘Well, of course they miss their friends, but they are so grateful for the time we are spending together that it makes up for the inconvenience’—she replied, to my astonishment.

Finally, the day came when they let us out. Wrapped in magic, that day dawned sunny, inviting us to leave the confinement behind. The 1-hour walk was not long in coming, but it was more than those pseudo-criminal walks that we took lately to the dumpster to throw away the rubbish and escape from the confinement for a minute. It was more than just the moment of applause when we looked at the door of the house to recognize the work of the health workers and that the children took advantage of it to see the neighbours’ children face-to-face, from the opposite pavement, and to run around, outside the law and up and down the street. Gone were those brief occasions of catharsis, which I now remember as endearing, in which we used to sing the famous Resistiré in unison while the children accompanied us by pounding their toy musical instruments and making music with the noise.

Leaving the house was a little ray of light at the end of the tunnel. It turned pale when we realized the conditions in which this tiny freedom was possible. Meeting friends and neighbours in the street and not being able to go near them to play or see them without a mask was hard for the children. My eldest son, who was very responsible, provoked a somewhat comical situation when he returned home from a walk with his father and gave vent to the indignation he felt after having seen that not everyone kept their distance, wore a mask or tried to prevent his sons and daughters from ‘mixing’ with those of other families. The little boy especially missed his best friends, some of whom he did not see on walks because, according to their parents, they were ‘so comfortable and happy at home with their brothers and sisters that they did not want to go out.’ Others did not go out at all. It is still too early to determine what mark all this will leave on us, especially the youngest.

Luckily, we have gradually been ‘de-escalating’ towards a ‘new normality’. It is scary to think whether it will be definitive. This will probably, perhaps recklessly, allow us to reopen the schools in September. I, for one, cannot wait. I need to recover the routine lost during the last 6 months but, above all, I believe that my children need to return to the more socializing space provided by the world we live in. This long impasse that they have experienced has perverted an interaction with peers in a normalized context that is essential for them. I cannot help but think that the world we adults have created is stealing childhood from the little ones and condemning them to a life of silence. Perhaps the goddess of fortune is asleep.

The measures that politicians are taking are far removed from the interests of children. The political class, rooted in its own particular realm with no one to oppose it, has narrowed its social horizons to such an extent that it has come to believe that it knows everything there is to know about the world and children. They cling to the idea of continuing to dictate the guidelines by turning a deaf ear to the needs of children. They have settled for the indifference, routine and stubbornness of those who do not know what to do but tell others what to do. The laws are made for one purpose only: to keep us at bay when our desires become excessive. But the desires of the little ones are not at all immoderate, and no law is needed to limit their most basic needs. The case of a guard dog that has been lovingly raised and yet confined for its entire life comes to mind. When he escapes from his cell, the world seems so strange to him that he begins to growl and become aggressive so that from then on he is branded as aggressive and spends the rest of his days in chains. The same thing can happen to children: the unnatural situation of being confined to their homes without being able to go out or see their friends and family can lead to them losing that loving and supportive impulse that must characterize human beings. If this situation continues over time, it is possible that they will irremissibly lose their path to socialization with others. I see it in adults, that mistrust of others as if they were the enemy, the infected. Children see it too and learn from what they are seeing. Perhaps it is necessary to cultivate a certain ignorance, a certain blindness, which allows for the gesture of the friend without any concealment, without fear. Without this blindness, life in society may become unbearable.

## Conclusions

As far as we know, this is the first study that explores the experiences of Spanish mothers and fathers of children during the pandemic. It has identified 13 themes that have formed the categories on which the story has been built.

Among these topics, the difficulties expressed by the participants to be able to combine their working life with the family and school life of the children stand out. In our story, we highlight the over-exertion that this has meant for the parents, more so considering that the period of confinement has turned out to be a period of school overload for the children (González-Calvo, 2020). These difficulties have been increased in those cases where work and health uncertainty surrounded the family environment, increasing the levels of depression, anxiety and stress suffered by adults (Wu et al., 2020), having to make real efforts to adapt and change in order to overcome the difficulties of the new situation. On the other hand, it is also true that some families have faced the situation from a more optimistic perspective that pretends to have its place in the narrative. Thus, the possibility of spending more time at home with the family, of taking care of the children and of teleworking has been seen as advantageous in the first place. They have sought to establish a family routine that included time for homework, time for physical exercise at home and the teaching of ‘important life lessons’. Being able to spend more family time has been, for many participants, a benefit of the pandemic, in the same vein as other studies (e.g. Fegert, Vitiello, Plener, & Clemens, 2020; González-Calvo, 2020). In line with other studies, participants in our research have sought to use pandemic time and confinement to experience meaningful situations and important learning for children (Coyne et al., 2020; Weaver & Swank, 2020).

Emotional distress and the high emotional impact of the health situation is another recurrent theme of our study. In addition to the positive aspects already mentioned (e.g. spending more time with the family), there are other negative aspects such as anxiety, worry and social isolation (Golberstein, Wen, & Miller, 2020; Patrick et al., 2020).

It is too early to ask whether COVID-19 is the disruptor of social change and transformation. What does seem clear is that, at least in the short and medium term, the virus will bring about a drastic change in our way of life: the way we relate to others, life in schools and leisure activities, among others, will become completely different (Lichfield, 2020).

In sum, we wanted to investigate, by asking the families of a group of children aged seven and eight, what vision they have about life during the pandemic and how they believe it will be the same in the near future. The most immediate finding of this research is the confirmation that the confinement of the children in the homes and the social distancing from their loved ones (friends and close relatives) have raised fears and concerns among mothers and fathers and, consequently, among the children. The worries, insecurities and deaths attributed to the virus, among other causes, have awakened feelings of anxiety and social phobia in families even though we cannot be sure yet to what extent. Likewise, they try to face the near future with hope and optimism, with the illusion that the situation will be as they remember it before the virus entered their lives. As the children’s families relate, the desire to live, to return to the parks, to hug friends, to play in groups and to be in school are among their greatest desires and needs.

As for the limitations of our study, it is worth noting the lack of diversity of the participants. Our research has involved families belonging to a middle social and economic class, many of them living in spacious houses with a garden that has made possible certain physical activities and contact with the natural environment that have facilitated the time of confinement, while in most cases, they have had access to the internet and technological resources to adequately follow school routines.

Finally, we would like to point out the importance of investigating and understanding how the families of minor children have experienced and lived an unprecedented event such as the health crisis due to COVID-19. Our study and the narrative through which we have tried to give voice to the parents of the children reveal the unique challenge that both adults and children have gone through in trying to adapt to what has been called the ‘new normal’. In this sense, we would like to point out the importance of our study in that it favours attentive listening to those who are normally ‘left without a voice’: mothers and fathers as ‘speakers’ for the youngest. Knowing how to listen to their concerns, their experiences, their fears and expectations about a health crisis situation – or any other – is important to be able to be understanding with them and help them overcome the hard process of adapting to a new life. Moreover, the emotional, physical and social well-being of people are at the core of close and active listening (Perrin, Leslie, & Boat, 2016). The voices of these participants, thus understood, can provide key elements for social, cultural, family and educational reconstruction after the COVID-19 crisis.
